# Molecular Status of *BRAF* Mutation in Epithelial Ovarian Cancer: An Analysis of 57 Cases in the Northeast of Iran

**DOI:** 10.30699/IJP.2023.554750.2907

**Published:** 2023-06-20

**Authors:** Amirhossein Jafarian, Masoumeh Jafaripour, Masoumeh Gharib, Maryam Salehi, Nema Mohamadian Roshan, Sare Etemad, Khatoone Mirshekar, Maryam Sheikhi, Masoumeh Heidari, Behnaz Ahmadian, Zahra Khoshnegah, Hossein Ayatollahi, Payam Siyadat

**Affiliations:** 1 *Cancer Molecular Pathology Research Center, Mashhad University of Medical Sciences, Mashhad, Iran*; 2 *Department of Surgical and Clinical Pathology, Faculty of Medicine, Mashhad University of Medical Sciences, Mashhad, Iran*; 3 *Clinical Research Unit, Mashhad University of Medical Sciences, Mashhad, Iran*; 4 *Department of Pathology, Mashhad University of Medical Sciences, Mashhad, Iran*; 5 *Department of Medical Genetics, School of Medicine, Yazd University of Medical Sciences, Yazd, Iran*; 6 *Department of Hematology, School of Allied Medical Sciences, Iran University of Medical Sciences, Tehran, Iran*

**Keywords:** BRAF V600E mutation, Direct sequencing, Epithelial ovarian cancer

## Abstract

**Background & Objective:**

Epithelial ovarian cancer (EOC) is the most prevalent type of ovarian cancer. Previous studies have elucidated different pathways for the progression of this malignancy. The mutation in the B-Raf proto-oncogene, serine/threonine kinase (BRAF) gene, a member of the MAPK/ERK signaling pathway, plays a role in the development of EOC. The current study aimed to determine the frequency of the BRAF V600E mutation in ovarian serous and mucinous tumors, including borderline and carcinoma subtypes.

**Methods:**

A total of 57 formalin-fixed paraffin-embedded samples, including serous borderline tumors (SBTs), low-grade serous carcinomas (LGSCs), high-grade serous carcinomas (HGSCs), mucinous borderline tumors (MBTs), and mucinous carcinomas, and 57 normal ovarian tissues were collected. The BRAF V600E mutation was analyzed using polymerase chain reaction (PCR) and sequencing.

**Results:**

While 40% of the SBT harbor *BRAF *mutation, we found no *BRAF *mutation in the invasive serous carcinoma (*P*=0.017). Also, there was only 1 *BRAF *mutation in MBT and no mutation in mucinous carcinomas. In addition, we found no mutation in the control group.

**Conclusion:**

The *BRAF *mutation is most frequent in borderline tumors but not in invasive serous carcinomas. It seems that 2 different pathways exist for the development of ovarian epithelial neoplasms: one for borderline tumors and the other for high-grade invasive carcinomas. Our study supports this hypothesis. The *BRAF *mutation is rare in mucinous neoplasms.

## Introduction

According to the World Health Organization (WHO), epithelial ovarian cancer (EOC) accounts for nearly 150,000 deaths per year worldwide (1, 2). EOC is a highly heterogeneous malignancy characterized by considerable genomic, morphologic, and clinical heterogeneity among different patients. Screening B-Raf proto-oncogene, serine/threonine kinase (*BRAF*) mutations can be important for the tumors’ classification and choosing appropriate treatment options (1, 3, 4). Previous studies have proposed a dualistic model for classifying EOCs into types I and II (5-7).

Type I tumors, including low-grade serous, clear cell, low-grade endometriosis, and mucinous carcinomas, are clinically slow-progressing tumors at a low stage at presentation. They exhibit an intermediate step between benign cystic neoplasms and associated carcinomas and can be regarded as borderline tumors. While significant morphological differences are present among type I tumors, the morphological differences among type II tumors are less apparent, with more overlapping features. Type II tumors diagnosed as high-grade serous and high-grade endometrioid display different patterns, including papillary, glandular, and solid (5, 8, 9).

The morphological differences between type I and II tumors can be related to marked distinctions in their molecular and genetic profiles. Type I tumors are genetically more stable than type II tumors; nearly two-thirds of patients with low-grade serous carcinoma (LGSC) harbor mutations in KRAS proto-oncogene, GTPase (*KRAS*), *BRAF*, and erb-b2 receptor tyrosine kinase 2 (*ERBB2*). However, tumor protein p53 (*TP53*) mutations are rather infrequent in these tumors (8, 10, 11).

*BRAF* p.V600E, a missense point mutation, is found in approximately 6% of ovarian cancers (12). Previous studies have shown the *BRAF* V600E mutation in more than 30% of serous borderline tumors (SBTs), which is associated with low malignant potential (LMP). However, it is rare in high-grade serous carcinomas (HGSCs) (13, 14). The present study aimed to determine the prevalence of the *BRAF* V600E mutation in a series of EOCs (low-grade, borderline, and invasive) in the northeast of Iran.

## Material and Methods

**Patients**

We collected 57 formalin-fixed paraffin-embedded samples of EOC tissues and 57 samples of normal tissues from the archived samples in the pathology laboratory of Qaem Hospital of Mashhad City, Iran. This study was approved by the Ethics Committee of Mashhad University of Medical Sciences (code: IR.MUMS.fm.rec.1395.471).

**Fig. 1:**
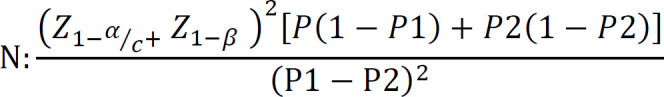
Sample size

The sample size is according to similar articles (15, 16) and the main purpose of the study (which is to compare the frequency in 2 groups). Considering the first- and second-type errors of 5% and 20%, respectively, at least 40 samples were determined in each group according to the following formula (Figure 1).

**P1=0.1, P2=0.35**

All samples were selected and reviewed by 2 experienced pathologists (M. J. J. and H. A.). The normal samples were analyzed from separate tissues that were non-tumor. The tumor samples consisted of HGSCs (n=30, 52.6%), mucinous borderline tumors (MBTs; n=7, 12.3%), LGSC (n=6, 10.5%), SBTs (n=5, 8.8%), mucinous carcinoma grade III (n=3, 5.3%), mucinous carcinoma grade II (n=2, 3.5%), mucinous carcinoma grade I (n=2, 3.5%), seromucinous carcinomas (n=1, 1.8%), and seromucinous borderline tumors. The normal samples included cases with luteal cysts (n=20, 35.1%) and normal ovarian tissues (n=37, 64.9%). 

**DNA Extraction**

DNAs were extracted using QIAamp DNA FFPE Tissue Kits (Cat No: 56404, Qiagen, Germany) according to the QIAGEN protocol. The quality and concentration of the DNAs were determined using a NanoDrop 1000 spectrophotometer (NanoDrop Technologies, Wilmington, DE, USA).

**Polymerase Chain Reaction Amplification and Direct Sequencing**

Polymerase chain reaction (PCR) was performed for the *BRAF* V600E mutation in a final volume of 25 μL, containing approximately 100 ng of genomic DNA, 12 μL of DW, 10 μL of master mix (Amplicon, Denmark), 100 nmol/L for primer forward 5′, TGCTTGCTCTGATAGGAAAATG -, 3′ and reverse 5′, AGCCTCAATTCTTACCATCCA -, 3′. The PCR amplification was performed by denaturation at 94°C for 5 min, followed by 35 amplification cycles at 94°C for 30 s, 58°C for 30 s, and 72°C for 30 s with an ultimate extension at 72°C for 30 s in a thermocycler (Applied Biosystems, USA). All PCR products were electrophoresed on a 2% agarose gel. Sequencing was performed for all samples, and the results were analyzed using a CLC sequence viewer. 

**Statistical Analysis**

Fisher’s exact test was used to evaluate correlations between the *BRAF* V600E mutation and the histopathological characteristics of tumors. *P* values less than 0.05 were considered statistically significant. SPSS version 16 (SPSS Inc, Chicago, IL, USA) was used for statistical analysis. 

## Results

**Clinicopathological Findings**

The clinicopathological data of all 114 cases, including 57 patients and 57 controls, were retrieved from their medical records; the pathological findings are summarized in Table 1. The median age was 48.51 ± 13.32 (range, 21-82) and 50.14 ± 13.64 (range, 25-81) for patients and controls, respectively. Also, the mean age of patients with serous, mucinous, and seromucinous ovarian tumors was 48.34 ± 13.1, 47.93 ± 15.06, and 56 ± 1.14 years, respectively. There was no statistically significant difference in age between patients and normal controls (*P*=0.52).

***BRAF*** **V600E Status**

**Fig. 2:**
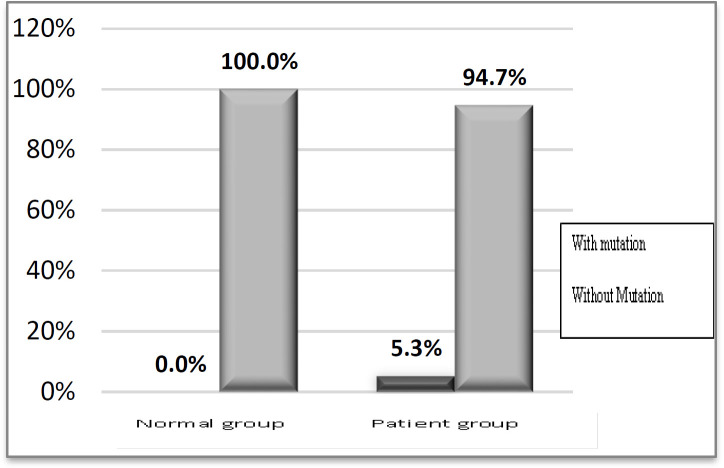
Frequencies of BRAF V600E mutation in Normal and Patient groups

**Fig. 3:**
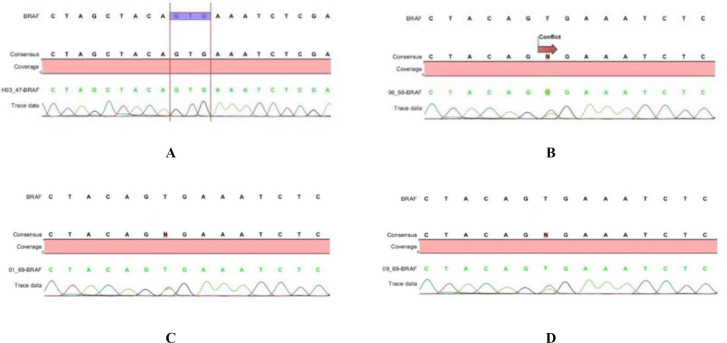
Chromatogram of the BRAF V600E mutation with direct sequencing method in surgical specimens from epithelial ovarian tumor patients. The A graph shows the wild-type sequence and the B graph shows BRAF V600E mutation in MBT patient. The C and D graph sequences show BRAF V600E mutation in SBT patients

**Fig. 4:**
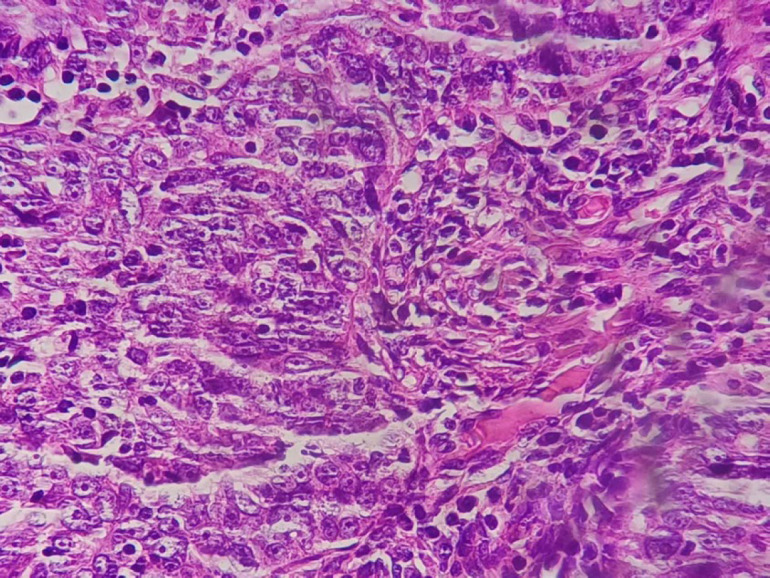
Atypical epitheloid cells with nuclear pleomorphism and cherry red nucleoli X400 in HIGH-GRADE papillary serous Carcinoma

**Fig. 5:**
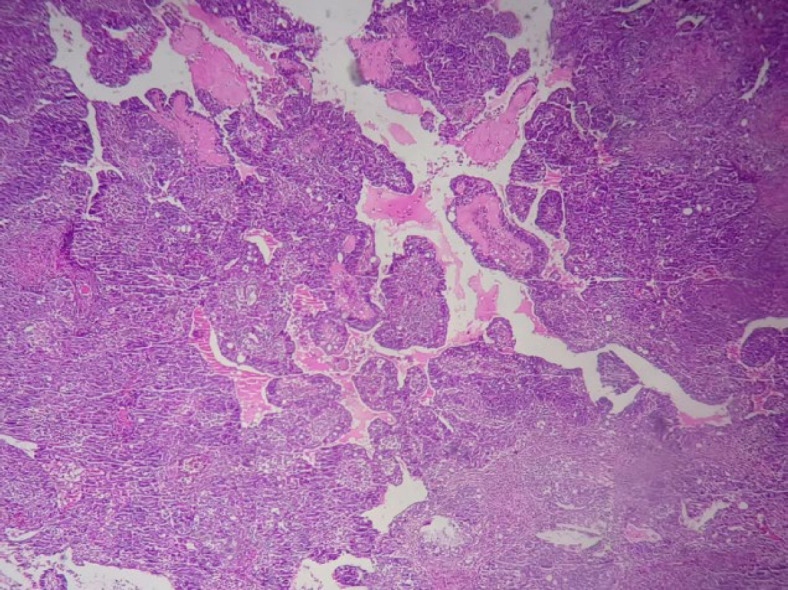
Slides show neoplastic proliferation of atypical epithelioid cells with high pleomorphism and cherry red nucleoli with papillary patern X100

**Fig. 6:**
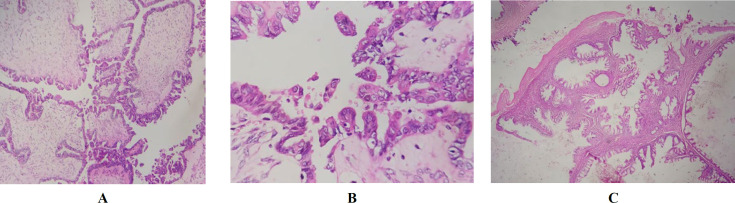
Slides showing neoplastic proliferation of atypical epithelioid cells with SBT (A,B) and MBT (C).

The *BRAF*V600E mutation was analyzed for both patient and control groups. As shown in Figure 2, the *BRAF* V600E mutation was present in 3 patients, including a patient with MBT and 2 patients with SBT. However, no one in the control group harbored this mutation (Figures 3, 4, 5, and 6). There were no significant differences in this mutation between the patient and control groups (*P*=0.24). In addition, no one with HGSC or LGSC carried the mutation. Furthermore, there were no significant correlations between the *BRAF* V600E mutation and the tumor histopathology in EOC patients (*P*=0.073; Table 1). Moreover, the frequency of the *BRAF* V600E mutation was not significantly different between mucinous and serous types of tumors (*P*>0.99; Table 1).

**Table 1:** Clinical Characteristics of 157 Patients with EOC. Tumors High Grade serous Carcinoma (HGSC), Mucinous Borderline Tumor (MBT), Low Grade Serous Carcinoma (LGSC), Serous Borderline Tumor (SBT)

Histopathology factors	BRAF V600E mutation: N + -	BRAF V600E mutation: %	P-value
HGSC MBT LGSC SBT	0 30 1 6 0 6 2 3	0% 14.28% 0 40%	*P*=0.073
Histologic grading mucinous carcinoma:			
FIGO III FIGO II FIGO I	0 3 0 2 0 2	0% 0% 0%	
Mucinous Type:			
Seromucinous carcinoma Mucinous Carcinoma Grade I	0 1 0 1	0% 0%	
Histologic type:			*P*>0.99
Serous Mucinous	2 39 1 13	4.87% 7.14%	
Histologic grading mucinous:			*P*>0.99
Borderline Carcinomas high grade	1 6 0 7	14.28% 0%	
Histologic grading serous:			*P*=0.017
Borderline Carcinomas high grade	2 3 0 30	40% 0	
Histologic grading serous:			*P*=0.182
Borderline Carcinomas low grade	2 3 0 6	40% 0	

## Discussion

Significant heterogeneity among different types of ovarian cancer, exhibiting different clinicopathological features, is regarded as one of the main challenges in understanding the exact mechanisms of this malignancy (17-20). While mucinous and endometrioid borderline tumors are frequently associated with invasive carcinomas, SBTs are rarely associated with serous carcinomas (2, 21).

Currently, detection of *BRAF* mutations is dependent on molecular methods, including direct sequencing, pyrosequencing, and amplification refractory mutation system PCR (ARMS-PCR). Also, the mutated *BRAF* protein has been particularly detected in tissues by the immunohistochemical staining method. The immunohistochemical expression of the VE1 protein is intensely dependent on the *BRAF* V600E mutation. If the immunohistochemical staining method was found appropriately, very sensitive and specific parallel determined with molecular techniques and are beneficial for tissues with low neoplastic cells (22). 

Previous studies have suggested *BRAF* V600E as an exclusive mutation in serous LMP and serous carcinomas. Besides, the latest studies have used the immunohistochemical method and shown a significant correlation between this mutation and a lower FIGO (International Federation of Gynecology and Obstetrics) stage in invasive carcinomas (13). 

Various studies (such as Wong *et al.*) using direct Sanger sequencing suggested a better clinical outcome for EOC patients with a *BRAF* mutation (12). Preusser *et al.* revealed that patients with invasive carcinomas stained positive for a *BRAF* V600E monoclonal antibody had a more favorable survival rate (13). In addition, other studies have demonstrated that the *BRAF* mutation is associated with serous tumors of a low malignant nature (12).

According to the previous studies, *BRAF* mutation was present in 23% to 71% of patients with SBT. However, this mutation was relatively infrequent in LGSC patients (18, 19). While *BRAF* mutation was infrequent in MBTs (2%), it was relatively more common in mucinous carcinoma and serous adenoma (14, 23-25). 

In the present study, we found the *BRAF* V600E mutation in 40% of SBT patients. However, there was no mutation in LGCS and HGSC groups. Our findings suggested a significant difference in the *BRAF* status between SBT and HGSC patients, whereas no significant difference was found between SBT and LGSC groups. Consistent with our results, Siben and colleagues reported a *BRAF* mutation in 36% of SBT patients and found no *BRAF* mutation in HGSC cases. Sequencing PCR was performed to detect the mutation (14).

In addition, Grishman *et al.* suggested the presence of the *BRAF *V600E mutation in SBT and LGS ovarian tumors with early-stage disease and a better clinical outcome (26). Bösmüller *et al.* reported a *BRAF* mutation in 71% and 14% of SBT and LGSC patients, respectively. Also, they found no *BRAF* mutation in HGSC cases (22). In 2010, Wong and colleagues found a *BRAF* mutation in 47% and 2% of SBT and LGSC patients, respectively. They proposed that the low frequency of a *BRAF* mutation in LGSC patients could be indicative of LGSC derivation from SBTs without a *BRAF* mutation (23). In 2015, they suggested that a *BRAF* mutation prevented tumor progression toward invasive stages and hence was relatively rare in advanced LGSC (12).

These findings suggest that LGSC cases without a *BRAF* mutation may progress into advanced stages. There was no significant difference in *BRAF* mutations between SBT and LGSC groups. Therefore, it is likely that some borderline tumors can progress into low-grade invasive carcinomas. On the contrary, SBT and HGSC groups showed significant differences in the *BRAF* status, suggesting the role of different pathways in their pathogenesis. Our findings also support the hypothesis that borderline serous and HGSC develop via different pathways. 

Furthermore, we found no statistically significant difference in *BRAF* mutations between serous and mucinous tumors (4.9% vs. 7.1%, respectively). While most studies have found *BRAF* mutation in serous tumors, it has been reported to be less infrequent in mucinous tumors (22, 27, 28).

Xu *et al.* suggested that *BRAF* mutations were frequent in borderline tumors. However, they showed no *BRAF* mutation in invasive serous carcinoma (29). It seems different pathways are involved in the pathogenesis of EOCs, and mechanisms that underlie borderline tumors are probably distinct from high-grade invasive carcinomas. Furthermore, some papers have suggested that due to the tumor's resistance to cytotoxic chemotherapy, patients with this mutation may benefit from *BRAF* inhibitors (30).

## Conclusion

Screening BRAF mutations may be helpful in classifications of ovarian tumors and selection an appropriate treatment. One of the main limitations of the current study was its small sample size. Therefore, more well-designed studies with larger sample sizes are needed to shed more light on the current issue, especially studies with focus on the effects of this mutation on the survival rate and prognosis of ovarian cancers.

## Funding

This research was funded through the MASHHAD University of Medical Science (grant number: 940392).

## Conflict of Interest

There were no conflict interest in this study.

## Ethics Approval & Consent to Participate

The study protocol was reviewed by the ethical committee of Mashhad University of Medical Sciences (IR.MUMS.fm.rec.1395.471).
